# The effects of *in ovo* administration of encapsulated Toll-like receptor 21 ligand as an adjuvant with Marek’s disease vaccine

**DOI:** 10.1038/s41598-018-34760-6

**Published:** 2018-11-06

**Authors:** Jegarubee Bavananthasivam, Leah Read, Jake Astill, Alexander Yitbarek, Tamiru N. Alkie, Mohamed Faizal Abdul-Careem, Sarah K. Wootton, Shahriar Behboudi, Shayan Sharif

**Affiliations:** 1 Department of Pathobiology, Ontario Veterinary College, University of Guelph, Guelph, Ontario, N1G 2W1 Canada; 2 Present Address: Department of Biology, Wilfred Laurier University, Waterloo, Ontario N2L 3C5 Canada; 3 Department of Ecosystem and public health, Faculty of Veterinary Medicine, University of Calgary, Calgary, Alberta T2N 1N4 Canada; 4 The Pirbright Institute, Ash Road, Pirbright, Woking, Surrey, GU24 0NF UK; 5 Department of Pathology and Infectious Disease, School of Veterinary Medicine, University of Surrey, Guildford, United Kingdom

## Abstract

Marek’s Disease Virus (MDV) is the causative agent of a lymphoproliferative disease, Marek’s disease (MD) in chickens. MD is only controlled by mass vaccination; however, immunity induced by MD vaccines is unable to prevent MDV replication and transmission. The herpesvirus of turkey (HVT) vaccine is one of the most widely used MD vaccines in poultry industry. Vaccines can be adjuvanted with Toll-like receptor ligands (TLR-Ls) to enhance their efficacy. In this study, we examined whether combining TLR-Ls with HVT can boost host immunity against MD and improve its efficacy. Results demonstrated that HVT alone or HVT combined with encapsulated CpG-ODN partially protected chickens from tumor incidence and reduced virus replication compared to the control group. However, encapsulated CpG-ODN only moderately, but not significantly, improved HVT efficacy and reduced tumor incidence from 53% to 33%. Further investigation of cytokine gene profiles in spleen and bursa of Fabricius revealed an inverse association between interleukin (IL)-10 and IL-18 expression and protection conferred by different treatments. In addition, the results of this study raise the possibility that interferon (IFN)-β and IFN-γ induced by the treatments may exert anti-viral responses against MDV replication in the bursa of Fabricius at early stage of MDV infection in chickens.

## Introduction

Marek’s disease (MD) in chickens is caused by an alpha herpesvirus, Marek’s Disease Virus (MDV), which is currently controlled in chicken farms by vaccination, selection of genetic lines resistant to MD and biosecurity measures. Depending on the host and virus virulence, MDV can cause more than 90% morbidity and mortality in susceptible chickens^1^. Chickens become infected by MDV following inhalation of infected dust via the respiratory route. MDV primarily infects macrophages, B and T cells and it mainly transforms CD4+ T cells although other T cell subtypes are susceptible to transformation^2,3^. Generally, MDV life cycle can be divided into the cytolytic phase (2–7 days post-infection - dpi), latent phase (7–10 dpi), late cytolytic phase, and transformation phase^2^. Lysis of lymphocytes and activation of TGF-beta + regulatory T cells are a few mechanisms among others which induce immunosuppression during MDV infection^4^. Furthermore, transformed T cells proliferate form lymphoma which can lead to immunosuppression and clinical and pathological signs of disease. In the course of infection, MDV replicates in feather follicular epithelium and sheds into the environment throughout the lifespan of an infected chicken.

Since MDV was first identified more than 50 years ago, several vaccines have been developed to control clinical signs of the disease, although none of them can fully prevent replication or transmission of MDV. Because of the inability of vaccines to control MDV transmission, it is believed that vaccines have prompted the emergence of virulent strains of MDV^5^. Herpesvirus of turkey (HVT) is one of the MD vaccines which is extensively used worldwide either alone or in combination with other MD vaccine^6^. HVT can be administered *in ovo* at embryonic day 18 (ED18) to provide protection against MDV in young chicks. However, protection offered by HVT is inadequate especially against very virulent and very virulent plus pathotypes of MDV^7^. Moreover, this vaccine will not be protective against emerging highly virulent strains of MDV in the future. Therefore, it would be of interest to determine whether efficacy of HVT can be enhanced by addition of immune stimulants.

Innate and adaptive defense mechanisms are necessary to control MDV infection in chickens. In young chicks, it is likely that *in ovo* administered vaccines initially confer protection mainly via innate immune system cells including Natural Killer (NK) like cells and macrophages rather than adaptive immune responses^8–11^. In general, the activation of innate responses orchestrates the induction of adaptive responses which in turn require several days to provide protective immunity against an infection. However, this is not well understood in regard to MD vaccination. In general, the immune system in young chicks undergoes maturation and becomes functionally mature around 1–2 weeks of age. Therefore, the induction of adaptive immune responses might be limited in the early stage of life and immediately after hatching. It has been reported that chicken T cells exhibit poor responsiveness to mitogen stimulation and are functionally immature during the first 2 weeks of age^12^. In addition, although it has been shown that chickens start to mount antibody-mediated immune responses at 1 week of age, they usually display higher levels of antibody production when they are immunized around 2 weeks of age^13,14^. There is a paucity of information on how MD vaccine modulates innate responses in young chickens. The constitutive expression of IFN-γ and IL-18 that is measurable from ED12 and higher expression of these cytokines by day 7 post-hatch^15^ indicates that innate responses may be functional prior to hatching. As a result, these responses can be exploited to accelerate the maturation of innate and adaptive immune responses. Increased IFN-γ expression observed with *in ovo* HVT vaccination in chickens^16^ as well as increased NK like activity observed with HVT vaccination at hatch and post-hatch^17,18^ revealed that innate responses can be elevated to significant level with appropriate immune modulations^19^. In addition, it has recently been reported that *in ovo* administration of HVT vaccine accelerates the maturation of the immune system by activation and expansion of T cells at hatch^20^.

TLR-Ls have been used experimentally as vaccine adjuvants or as stand-alone anti-viral agents^21–24^. For instance, TLR3 ligand, polyinosinic:polycytidylic acid (Poly(IC)), has been shown to induce innate responses in chickens^25^ and its administration to chickens combined with HVT could lead to reduction of tumor incidence after challenge with a very virulent strain of MDV^26^. In addition, TLR4 ligand, lipopolysaccharide (LPS), and CpG-ODN, the ligand for TLR21 have been shown to stimulate innate responses mainly via the induction of interferons (IFNs) and interleukin (IL)1-β and, which may lead to a reduction in MD progression in chickens^27^. Prolonged availability of TLR-Ls in a controlled-release fashion which can be achieved by encapsulation in poly(D, L-lactic-co-glycolic) acid (PLGA) nanoparticles has been shown to induce innate responses *in vitro*, *in ovo* and *in vivo*^19,28^. Administration of encapsulated CpG (ECpG) as a prophylactic agent demonstrated considerable effects on the outcome of MD in our previous study^29^, which prompted further investigation of the adjuvant effect of ECpG with MDV vaccine in chickens.

The present study was designed to study the adjuvant effect of ECpG in conjunction with HVT vaccine administered either *in ovo* to ED18 embryos or to chickens 14 days after an experimental infection with MDV or at both time points to improve immunity against MDV infection in chickens.

## Results

### MDV tumor incidence

**Figure 1:**
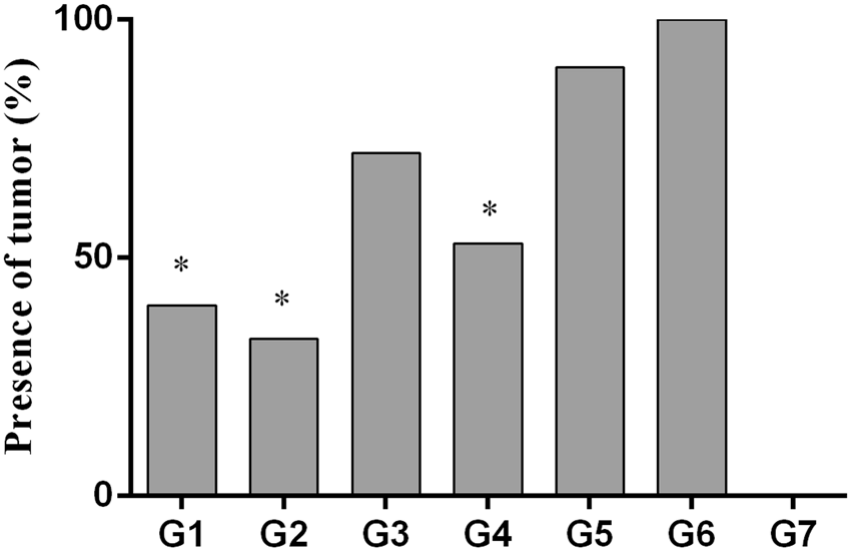
Presence of tumors in different treatment groups. Experimental groups were as follows: G1-ECpG and HVT were administered at ED18 and the second dose of ECpG was injected at 14 dpi. G2-ECpG and HVT were administered at ED18. G3-HVT was administered at ED18 and ECpG was given at 14 dpi. G4-HVT was administered at ED18. G5-ECpG was injected at 14 dpi. G6-Untreated, MDV-infected group. G7-PBS control group. Chickens in all groups were infected with MDV at d5 except G7. Presence of tumors was recorded at 21 dpi. Data were statistically analyzed by Fisher’s exact test in GraphPad Prism 6.04. *p* ≤ 0.05 (*) was considered statistically significant compared to G6.

Treatment groups were compared to the positive control group (G6), in which all chickens developed tumors (Fig. 1, Supplementary Table 1). Chickens in group 1 that received ECpG and HVT at ED18, and ECpG again at 14 dpi showed 40% tumors which was statistically significant compared to positive control chickens (*p* = 0.002, Fig. 1). Chickens in group 2 which received ECpG and HVT at ED18 developed the lowest tumor incidence compared to positive control chickens (33.33%, *p* = 0.001). In HVT only group (G4), 53.33% of the chickens exhibited tumors, which was significantly different compared to positive control chickens (*p* = 0.01, Fig. 1). In G3 and G5, 72.72% and 90% of the chickens developed tumors respectively (Fig. 1). There was no statistically significant difference in tumor incidence between chickens in G4 and ECpG administered groups (G1, G2 and G3). However, the highest reduction in tumor incidence was observed in chickens in G2 (Fig. 1). Therefore, it seems that ECpG partially improved the HVT induced protection against MD.

### MDV genome copy numbers in feathers

**Figure 2:**
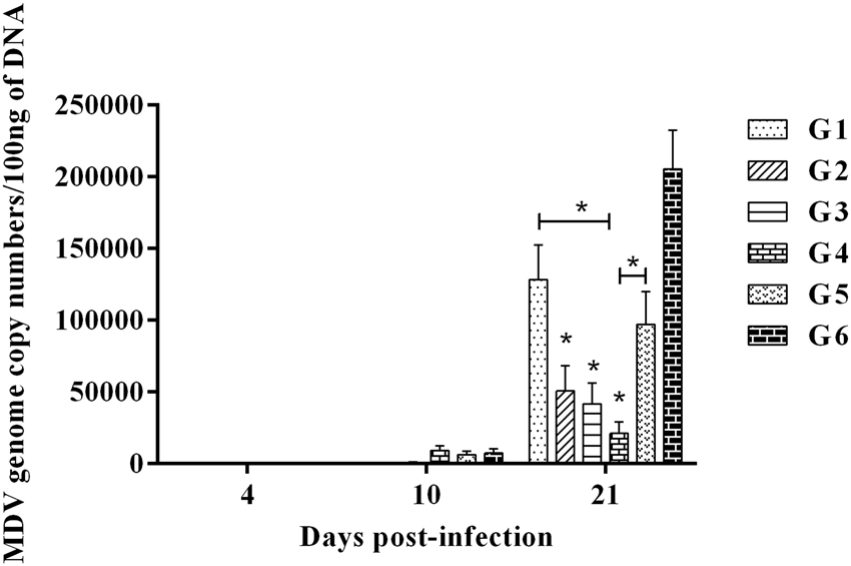
MDV genome copy numbers in feathers in different treatment groups. MDV genome copy numbers per 100 ng of DNA were calculated from feathers collected at 4, 10 and 21 dpi. The different experimental groups were: G1-ECpG and HVT were administered at ED18 and the second dose of ECpG was injected at 14 dpi. G2-ECpG and HVT were administered at ED18. G3-HVT was administered at ED18 and ECpG was given at 14 dpi. G4-HVT was administered at ED18. G5-ECpG was injected at 14 dpi. G6-Untreated, MDV-infected group. G7-PBS control group. Chickens in all groups were infected with MDV at d5 except G7. Data were statistically analyzed by Kruskal-Wallis test in GraphPad Prism 6.04. *p* ≤ 0.05 was considered statistically significant (*). Comparisons were made between groups as indicated or with positive control group G6. Error bars indicate the standard errors of the mean.

At 4 dpi, there was no genome detected in feather DNA. MDV load gradually increased throughout the experiment and was at the highest level at 21 dpi (Fig. 2). Chickens in group 2, 3 and 4 had significantly lower MDV load in feathers at 21 dpi when compared to that of G6 (*p* < 0.0001, Fig. 2). In addition, significantly lower MDV load was detected in G4 chickens when compared to G1 and G5 chickens (*p* < 0.0001, Fig. 2). Unfortunately, administration of ECpG was unable to enhance the HVT induced reduction of MDV load in feathers.

### Organ weight indices

**Figure 3:**
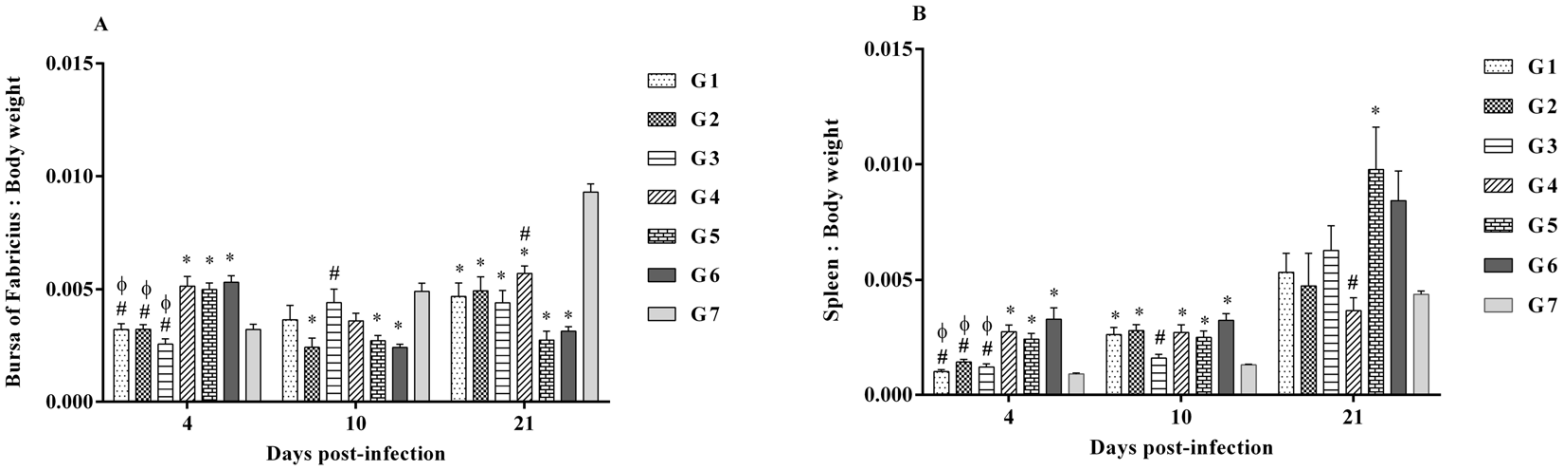
BF:BW and spleen:BW ratios in different treatment groups. BF, spleen and body weight were recorded at 4, 10 and 21 dpi, and (**A**) BF:BW and (**B**) Spleen:BW ratios were calculated. The different experimental groups were: G1-ECpG and HVT were administered at ED18 and the second dose of ECpG was injected at 14 dpi. G2-ECpG and HVT were administered at ED18. G3-HVT was administered at ED18 and ECpG was given at 14 dpi. G4-HVT was administered at ED18. G5-ECpG was injected at 14 dpi. G6-Untreated, MDV-infected group. G7-PBS control group. Chickens in all groups were infected with MDV at d5 except G7. Data were statistically analyzed by one-way ANOVA and followed by Tukey’s multiple comparison test in GraphPad Prism 6.04. *p* ≤ 0.05 was considered statistically significant when compared to G7 (*) or G6 (#) or G4 (ϕ). Error bars indicate the standard errors of the mean.

At 4 dpi, bursa of Fabricius weight to body weight (BF:BW) ratios were significantly higher in G4, G5 and G6 chickens when compared to the chickens in the PBS control group (G7) (*p* < 0.0001, Fig. 3A). Although at 4 dpi BF:BW ratios in G1, G2 and G3 chickens were almost similar when compared to G7 chickens, they were significantly different from that of G6 chickens (*p* < 0.0001, Fig. 3A). At 10 dpi, G2, G5 and G6 chickens showed significantly lower BF:BW ratios when compared to G7 chickens *(p* = 0.0001). However, G3 chickens exhibited significantly higher ratios when compared to G6 chickens (*p* = 0.0001, Fig. 3A). At 21 dpi, chickens in all MDV-infected groups showed significantly lower BF:BW ratios when compared to G7 chickens (*p* < 0.0001, Fig. 3A). However, the ratio was significantly higher in G4 when compared to G6 (*p* < 0.0001, Fig. 3A). This indicates that HVT vaccine can partially inhibit bursal atrophy induced by MDV.

At 4 dpi, spleen:BW ratios were significantly higher in G4, G5 and G6 chickens when compared to G7 chickens and ratios were significantly lower in G1, G2 and G3 chickens when compared to G6 chickens (*p* < 0.0001, Fig. 3B). At 10 dpi, chickens in all groups which received MDV except G3 had significantly higher spleen:BW ratios when compared to G7, and G3 chickens had significantly lower spleen:BW ratios when compared to G6 chickens (*p* < 0.0001, Fig. 3B). At 21 dpi, G5 chickens showed significantly higher spleen:BW ratio when compared to G7 chickens (*p* < 0.0015, Fig. 3B). However, G4 chickens showed significantly lower ratio when compared to G6 chickens (*p* < 0.0015, Fig. 3B).

### Cytokine gene expression

**Figure 4:**
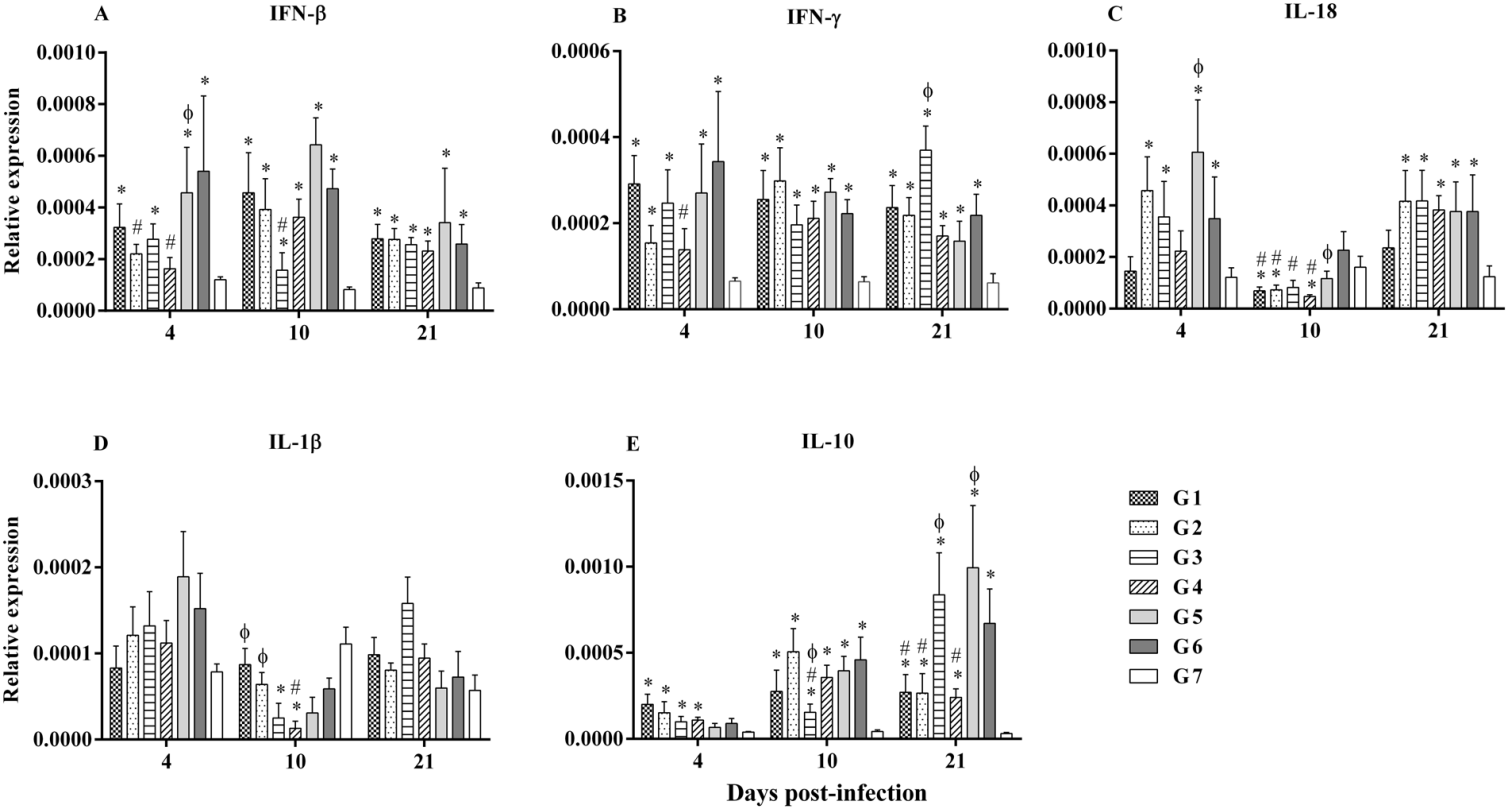
Relative expression of genes in spleen in different treatment groups. (**A**) IFN-β (**B**) IFN-γ (**C**) IL-18 (**D**) IL-1β and (**E**) IL-10 expression were determined relative to β-actin at 4, 10 and 21 dpi in spleen. The different experimental groups were: G1-ECpG and HVT were administered at ED18 and the second dose of ECpG was injected at 14 dpi. G2-ECpG and HVT were administered at ED18. G3-HVT was administered at ED18 and ECpG was given at 14 dpi. G4-HVT was administered at ED18. G5-ECpG was injected at 14 dpi. G6-Untreated, MDV-infected group. G7-PBS control group. Chickens in all groups were infected with MDV at d5 except G7. Data were logarithmically transformed and analyzed using general linear model (Proc GLM) and followed by Duncan’s multiple range test in Statistical Analysis Software version 9.3 (SAS, Cary, NC). The Kruskal-Wallis test was used when data were not normally distributed. Gene expression results were presented as geometric mean of relative expression ± standard error of mean. *p* ≤ 0.05 was considered statistically significant when compared to G7 (*) or G6 (#) or G4 (ϕ).

In spleen, IFN-β was significantly upregulated at all time points in all MDV-infected groups except G2 and G4 at 4 dpi when compared to G7 (*p* ≤ 0.001, Fig. 4A). Similarly, IFN-γ was significantly upregulated at 4, 10 and 21 dpi in all MDV-infected groups, except G4 at 4 dpi, compared to G7 (*p* ≤ 0.001, Fig. 4B). IL-18 was significantly upregulated in G2, G3, G5 and G6 at 4 dpi, whereas this cytokine was downregulated in G1, G2 and G4 at 10 dpi. Again, at 21 dpi, IL-18 was upregulated in G2, G3, G4, G5 and G6 when compared to G7 (*p* ≤ 0.025, Fig. 4C). Although there was no significant upregulation of IL-1β at any time point, slightly higher expression of IL-1β was observed in G1, G3 and G4 chickens at 21 dpi when compared to G6 (Fig. 4D). IL-10 expression was significantly upregulated in the chickens in all the groups that received HVT at 4 dpi (*P* = 0.007) as well as in MD-infected groups at 10 and 21 dpi (*p* < 0.0001) when compared to G7 chickens (Fig. 4E). Noticeably, IL-10 expression was significantly lower in G3 chickens at 10 dpi and G1, G2 and G4 chickens at 21 dpi when compared to the chickens in G6 (*p* < 0.0001, Fig. 4E).

**Figure 5:**
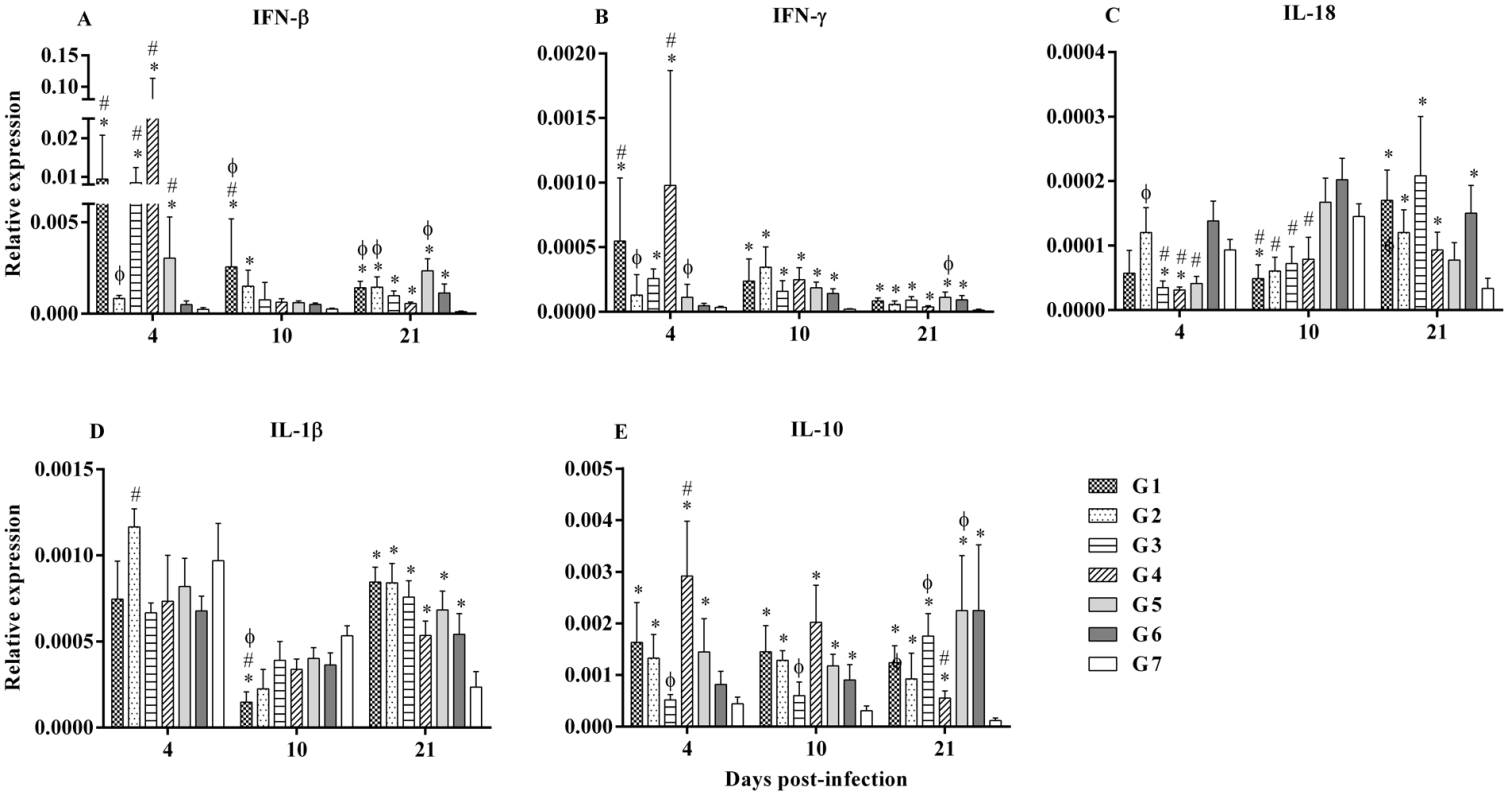
Relative expression of genes in the bursa of Fabricius in different treatment groups. (**A**) IFN-β (**B**) IFN-γ (**C**) IL-18 (**D**) IL-1β and (**E**) IL-10 expression were determined relative to β-actin at 4, 10 and 21 dpi in BF. The different experimental groups were: G1-ECpG and HVT were administered at ED18 and the second dose of ECpG was injected at 14 dpi. G2-ECpG and HVT were administered at ED18. G3-HVT was administered at ED18 and ECpG was given at 14 dpi. G4-HVT was administered at ED18. G5-ECpG was injected at 14 dpi. G6-Untreated, MDV-infected group. G7-PBS control group. Chickens in all groups were infected with MDV at d5 except G7. Data were logarithmically transformed and analyzed using general linear model (Proc GLM) and followed by Duncan’s multiple range test in Statistical Analysis Software version 9.3 (SAS, Cary, NC). The Kruskal-Wallis test was used when data were not normally distributed. Gene expression results were presented as geometric mean of relative expression ± standard error of mean. *p* ≤ 0.05 was considered statistically significant when compared to G7 (*) or G6 (#) or G4 (ϕ).

In the bursa of Fabricius, at 4 dpi, IFN-β was significantly upregulated in G1, G3, G4 and G5 chickens when compared to G6 and G7 chickens (*p* < 0.0001, Fig. 5A). At 10 dpi, IFN-β was significantly upregulated in G1 chickens when compared to G6 and G7 chickens, and in G2 when compared to G7 (*p* = 0.002, Fig. 5A). At 21 dpi, IFN-β was upregulated in all groups that were challenged with MDV (*p* < 0.0001, Fig. 5A). Further, IFN-γ was upregulated in chickens at all time points and in all the groups which received MDV when compared to chickens in G7 except the chickens in G2, G5 and G6 at 4 dpi (*p* ≤ 0.0002, Fig. 5B). Expression of IFN-γ was significantly upregulated in G1 and G4 chickens at 4 dpi when compared to G6 chickens (*p* ≤ 0.0002, Fig. 5B). Downregulation of IL-18 was observed in G3, G4 and G5 chickens at 4 dpi and also at 10 dpi in chicken in all the groups that received HVT when compared to G6 chickens (*p* ≤ 0.009, Fig. 5C). However, at 21 dpi, IL-18 was upregulated in chickens in all MDV-infected groups, except in G5 chickens, compared to G7 chickens (*p* = 0.001, Fig. 5C). Expression of IL-1β was upregulated in G2 chickens compared to G6 chickens at 4 dpi (Fig. 5D). IL-1β was upregulated at 10 dpi in G1 chickens and at 21 dpi in chickens in all the groups that received MDV when compared to G7 chickens (*p* ≤ 0.04, Fig. 5D). Expression of IL-10 was upregulated in the chickens in all MDV-infected group at 4, 10 and 21 dpi compared to G7 chickens except G3 and G6 at 4 dpi and, G3 at 10 dpi (*p* ≤ 0.0005, Fig. 5E). IL-10 was significantly higher at 4 dpi and lower at 21 dpi in G4 chickens compared to G6 chickens (*p* ≤ 0.0005, Fig. 5E).

## Discussion

To enhance host responses against MDV, administration of ECpG along with HVT was investigated in the current study. Among the HVT-administered groups, a significant reduction in both tumor incidence and MDV load in feathers was observed in G2 and G4 chickens. Chickens in G1 showed significant reduction only in tumors whereas chickens in G3 showed significant reduction only in MDV load in feathers. Although there was no statistically significant difference in tumor incidence between chickens in G4 and groups receiving ECpG (G1, G2 and G3), administration of ECpG along with HVT at ED18 appeared to have an additive effect to HVT vaccination, marked by the lowest tumor incidence in G2 chickens.

The observed reduction of MDV load in feathers of chickens treated with ECpG and HVT or HVT alone is similar to the reduction reported in a previous study using Rispens-CVI988 vaccine^30^, which is known to be the most effective vaccine against very virulent and very virulent plus MDV^31^. Since a reduction of MDV load in feathers was observed in the current study, further investigation of MDV transmission pattern in the environment especially in the dust and between chickens will answer whether this reduction interrupts the spread of MDV. Particularly, the use of resistant and susceptible genotypes of chickens may validate the use of ECpG with HVT as the virulence of MDV can be masked in resistant line of chickens^32^.

Commonly, MDV infection results in bursal atrophy due to B cell cytolysis and spleen enlargement is due to inflammation as well as tumor formation. Hence, organ weight indices were recorded to evaluate the changes occurring in BF and spleen following different treatments and MDV challenge in chickens. Administration of either ECpG and HVT or HVT alone possibly inhibited the initial cytolysis of B cells in BF as indicated by the BF:BW ratios similar to those of the control group. The observed abrogation of bursal atrophy is similar to the previous reports^33,34^. HVT administration with or without ECpG might have resolved the physiological changes which occurred in spleen at the initial stage of MDV infection since there was no difference in the ratios of spleen:BW between the chickens in HVT-administered group and uninfected group at 21 dpi. However, further investigation is needed to test this possibility.

The significant upregulation of the cytokines, IFN-β and IFN-γ, in BF at 4 dpi raises the possibility that the anti-viral activity of these cytokines might have repressed MDV replication at the initial stage of MDV pathogenesis and led to the reduction in tumor incidence and MDV load in feathers. Previous study indicated that involvement of IFN-β and IFN-γ in preventing DNA and virion synthesis in the case of herpes simplex virus type 1 (HSV1) infection^35^. Since MDV and HSV belong to the subfamily *alphaherpesvirinae*, we can speculate that IFN-β and IFN-γ may have similar functions for preventing the replication of MDV in infected cells. Another study also revealed that TLR-Ls induced IFN-β expression provide protection against HSV2 infection^36^. These lines of evidence and our findings support the role played by IFN-β induced by ECpG and HVT in immunity against MD in chickens. IFN-γ possibly exerts anti-viral effects on MDV-infected cells directly or indirectly via activation of macrophages^37^. Significant upregulation of IFN-γ at 4 dpi in BF in some treatment groups compared to G6 indicates initial anti-viral effects which might have contributed to the subsequent reduction of tumors in chickens. Overall, the expression profile of IFN-γ in spleen and BF shows the association of IFN-γ with immunity to MDV infection. This is in agreement with previous studies showing the importance of IFN-γ in HVT conferred immunity in chickens^38,39^.

IL-10 expression in chickens is associated with MD progression. It has been suggested that IL-10 curtails T helper (Th)1 immune responses, which are critical for protection, by deviating responses towards Th2 responses^40^. This could also be the case for MDV infection as an association between IL-10 expression and the disease was reported^41,42^. IL-10 affects dendritic cell (DC) maturation by inhibition of costimulatory molecules, DC induced T cell activation, antigen-specific T cell proliferation and function^43,44^; and it promotes the expansion of inducible T regulatory cells^45^. Notably, the experimental groups in the current study that exhibited significant reduction in tumor incidence displayed lower expression of IL-10 in spleen at 21 dpi. In addition, chickens that had tumors, expressed elevated levels of IL-10 in spleen and BF compared to those chickens that did not have tumors in each treatment group (data not shown). A similar pattern of IL-10 expression was recorded in previous studies in a different context, for example when chickens were vaccinated with bivalent or Rispens-CVI988 vaccine followed by MDV infection^30,41^. Therefore, previous reports were further confirmed and extended in the current study as the suppression of IL-10 was inversely associated with protection.

The other interesting finding in the current study was the downregulation of proinflammatory cytokines, particularly IL-18 expression in chickens in the treatment groups compared to G6 chickens. It has been noted previously that expression of IL-18 diminished in MD resistant lines of chickens^46^ compared to that of susceptible lines and also in vaccine protected chickens compared to unvaccinated-infected controls^39,41^. Similar observations were confirmed in the current study as indicated by the association between decreased expression of IL-18 and protection against MDV as shown by the reduction of tumor incidence and MDV load in feathers. Moreover, IL-18 supports the metastasis of melanoma cells via elevated expression of vascular cell adhesion molecule-1 (VCAM-1)^47^. Therefore, it is probable that decreased expression of IL-18 in the current study may have abrogated the expression of VCAM-1, which possibly contributed to the reduction of tumor incidence.

In conclusion, administration of ECpG with HVT provided protection against MDV in chickens by reducing tumor incidence and MDV loads in feathers. The anti-viral ability of IFN-β and IFN-γ might have contributed to the outcome of the treatments against MDV infection. In addition, downregulation of IL-10 and IL-18 by HVT and/or ECpG treatment appears to be linked to defense against MDV infection as demonstrated by the significant reduction of tumor formation and MDV load in feathers.

## Methods

### Chicken eggs, incubation and housing

Specific pathogen free (SPF) eggs that were obtained from the Animal Disease Research Institute, Canadian Food Inspection Agency (Ottawa, Ontario, Canada) were incubated at recommended temperature and relative humidity at the Arkell Research Station, University of Guelph. All experiments were approved by the Animal Care Committee of the University of Guelph and conducted according to the guidelines of the Animal Care Committee of the University of Guelph. At ED18, live embryos were administrated with ECpG or HVT or both into the amniotic sac using a 23-gauge, 2.5 cm needle as recommended. Once the chicks hatched, they were transported to the animal isolation facility at the Ontario Veterinary College, University of Guelph.

### TLR ligand, vaccine and virus

Synthetic Class B CpG ODN 2007 [5′-TCGTCGTTGTCGTTTTGTCGTT-3′] with phosphorothioate backbone was purchased from Sigma-Aldrich (Oakville, ON, Canada). FC-126 strain of HVT vaccine was received from Merial Canada Inc (Boehringer-Ingelheim Canada Ltd, Quebec, Canada). *In vivo* propagated, very virulent strain of MDV (RB1B) titrated in chicken kidney cells was used to infect chicks^48^.

### Encapsulation of TLR ligand

CpG ODN was encapsulated in PLGA, (Resomer® RG 503 H, free carboxylic acid, MW 24–38 kD, Sigma-Aldrich) using the modified double emulsion solvent evaporation method to generate PLGA nanoparticles as described in our previous studies^19,28^. Briefly, polyethalenimine (linear, MW 2.5 kD, Sigma-Aldrich) was complexed with CpG at a defined ratio^49^. This complex was layered onto 4.5% PLGA dissolved in dichloromethane (DCM, Sigma-Aldrich) and sonicated for one minute at 40% amplitude using an Ultrasonic Processor (Fisher Scientific). The resulting emulsion was sonicated with 2% polyvinyl alcohol (PVA)/1% poloxamer (PVA, MW 30–70 kD, 87–90% hydrolyzed, Sigma Aldrich) for two minutes at 60% amplitude and the resulting suspension was hardened by mixing with 50 mL of 2%PVA/1% poloxamer solution. The PLGA nanoparticles were harvested by centrifugation at 20,000 g for 50 min at 4 °C and washed three times with ultrapure nuclease free water. The PLGA nanoparticle suspension was frozen at −80 °C for 1–2 hours, then lyophilized for 18–22 hours (FreeZone®18 Liter Freeze Dry Systems, Labconco Corporation, Kansas City, Missouri) and sterilized as indicated^28^. Encapsulation efficiency was determined as in our previous studies^19,28^.

### Experimental design

Seven groups of ED18 chicken embryos (38–45 embryos per group) were injected via the amniotic route with approximately 25 µg ECpG and a quarter (1/4) of the recommended dose of HVT or HVT alone or phosphate buffered saline (PBS). Once the chicks hatched, at 5 days of age (5d), all chicks, except the PBS group were infected with 250 plaque-forming unit (PFU) of very virulent RB1B MDV per chick via intra-abdominal route. The untreated, but MDV-infected group was used as a positive control group. At 14 dpi, chickens were given a second dose of ECpG or PBS. Experimental groups in this study were designated as follows. Chickens in group 1 (G1) received ECpG and HVT at ED18, infected with MDV at day 5 (d5) and received a second dose of ECpG at 14 dpi. Group 2 (G2) chickens were administered with ECpG and HVT at ED18 and infected with MDV at d5. Chickens in group 3 (G3) were injected with HVT at ED18, infected with MDV at d5 and administered ECpG at 14 dpi. Group 4 (G4) chickens were administered with HVT at ED18 and infected with MDV at d5. Group 5 (G5) chickens received MDV at d5 and ECpG at 14 dpi. Group 6 (G6) chickens received MDV at d5. Chickens in group 7 (G7) received PBS. Each group had between 9–15 chickens at different time points. At 4, 10 and 21 dpi, spleen, and bursa of Fabricius weight (BF), and body weight (BW) were recorded and organ weight indices were calculated relative to the body weight of chickens. Samples from spleen, BF and feathers were collected in RNAlater and stored at −80 °C until further processing. Tumor incidence following infection with RB1B MDV was documented at 21 dpi.

### DNA and RNA extraction and cDNA synthesis

Genomic DNA was extracted from feather tips as described previously^50^. Feather tips were cut into small pieces and incubated with 500 µl cell lysis buffer (10 mM Tris, pH 7.5, 10 mM NaCl, 1 mM EDTA, pH 8 with 0.5% (w/v) Sarkosyl) containing 100 µl of proteinase K (10 mg/ml) overnight at 65 °C water bath. Extracted DNA was precipitated using 25 µl 5 M NaCl and 2.3 ml 95% ethanol. DNA concentration was measured and dilutions at a concentration of 50 ng/µl of DNA samples were prepared. A hundred nanograms of diluted DNA was used for MDV genome copy numbers quantification using quantitative real-time PCR.

RNA was extracted from spleen and BF using Trizol (Life Technologies, Burlington, Ontario) according to the manufacturer’s protocol. cDNA was synthesized from 1 µg of DNase treated RNA using Oligo_(dT)_12–18 primers and the Super-Script^TM^ First-Strand Synthesis System kit (Life Technologies, Burlington, Canada) according to manufacturer’s instructions. Synthesized cDNA was diluted at 1:10 in nuclease free water for evaluating the expression of cytokine genes. The quantity and quality of DNA and RNA were determined using the NanoDrop^®^ ND-1000 spectrophotometry (NanoDrop Technologies, Wilmington, DE).

### Real-time PCR

Real-time PCR was performed using SYBR green dye in a LightCycler 480 II to quantify the MDV genome copy numbers and cytokine gene expression (Roche Diagnostics, Laval, Quebec) as described previously^50,51^. Primer sequences of target and reference genes are listed in Table 1. The primers were synthesized by Sigma–Aldrich Canada (Oakville, ON).

**Table 1:** Target genes, primer sequences and references used for real-time PCR.

Genes	Primer sequences	References
β -actin	F: 5′-CAACACAGTGCTGTCTGGTGGTA-3′	^52^
	R: 5′-ATCGTACTCCTGCTTGCTGATCC-3′	
meq	F: 5′-GTCCCCCCTCGATCTTTCTC-3′	^50^
	R: 5′-CGTCTGCTTCCTGCGTCTTC-3?’	
IFN- β	F: 5′-GCCTCCAGCTCCTTCAGAATACG-3′	^53^
	R: 5′-CTGGATCTGGTTGAGGAGGCTGT-3′	
IFN-γ	F: 5′-ACACTGACAAGTCAAAGCCGC-3′	^54^
	R: 5′-AGTCGTTCATCGGGAGCTTG-3′	
IL-18	F: 5′-GAAACGTCAATAGCCAGTTGC-3′	^55^
	R: 5′-TCCCATGCTCTTTCTCACAACA-3′	
IL-1β	F: 5′-GTGAGGCTCAACATTGCGCTGTA-3′	^56^
	R: 5′-TGTCCAGGCGGTAGAAGATGAAG-3′	
IL-10	F: 5′-AGCAGATCAAGGAGACGTTC-3′	^41^
	R: 5′-ATCAGCAGGTACTCCTCGAT-3′	

### Statistical analysis

Relative target gene expression was calculated using chicken β-actin in LightCycler 480 II advanced relative quantification software. Data was logarithmically transformed and analyzed using general linear model (Proc GLM) in Statistical Analysis Software version 9.3 (SAS, Cary, NC). Duncan’s multiple range test was performed to determine significantly different groups. Gene expression results were presented as the geometric mean of relative expression ± standard error of mean. Tumor incidence data were analyzed by Fisher’s exact test (GraphPad Prism version 6.04). One-way ANOVA followed by Tukey’s multiple comparison test was used to analyze BF:BW and spleen:BW ratios, and MDV genome copy numbers. When data were not normally distributed Kruskal-Wallis test was used. Results were considered significant if *p* value was < 0.05.

## Electronic supplementary material

Supplementary  tables

## Data Availability

The datasets generated and analyzed during the current study are available from the corresponding author on reasonable request.
