# Supplementary material for: The effects of in ovo administration of encapsulated Toll-like receptor 21 ligand as an adjuvant with Marek’s disease vaccine
Source: Sci Rep. 2018 Nov 6;8:16370. doi: 10.1038/s41598-018-34760-6 (PMC6219601; doi:10.1038/s41598-018-34760-6)
Supplement: Supplementary file 1 — Supplementary tables [file 41598_2018_34760_MOESM1_ESM.docx]

**The effects of *in ovo* administration of encapsulated Toll-like receptor 21 ligand**

**as an adjuvant with Marek’s disease vaccine**

Jegarubee Bavananthasivam, Leah Read, Jake Astill, Alexander Yitbarek, Tamiru N. Alkie, Mohamed Faizal Abdul-Careem, Sarah K. Wootton, Shahriar Behboudi, Shayan Sharif

Table 1: Number of tissues in each bird harboring tumors as well as the number of sick birds showing clinical signs at 21 days post-infection (dpi)

| Groups | Presence of tumor (%) | Tumor containing tissues | Sick birds at 21dpi |
| --- | --- | --- | --- |
| G1 | 40 | 6 | 2 |
| G2 | 33 | 7 | 1 |
| G3 | 73 | 8 | 2 |
| G4 | 53 | 13 | 3 |
| G5 | 90 | 16 | 5 |
| G6 | 100 | 24 | 5 |

Table 2: Frequency of RB1B meq gene positive chickens over sampled chickens in each group at different time points

| Time point | Treatment groups | | | | | |
| --- | --- | --- | --- | --- | --- | --- |
|  | G1 | G2 | G3 | G4 | G5 | G6 |
| 4 dpi | 0/10 | 0/9 | 0/10 | 0/10 | 0/9 | 0/9 |
| 10 dpi | 4/10 | 5/10 | 3/9 | 7/10 | 10/10 | 10/10 |
| 21 dpi | 15/15 | 12/12 | 11/11 | 14/15 | 10/10 | 9/10 |
